# Ultrastructural Analysis of *Prune Dwarf* *Virus* Intercellular Transport and Pathogenesis

**DOI:** 10.3390/ijms19092570

**Published:** 2018-08-29

**Authors:** Edmund Kozieł, Katarzyna Otulak-Kozieł, Józef J. Bujarski

**Affiliations:** 1 Faculty of Agriculture and Biology, Department of Botany, Warsaw University of Life Sciences—SGGW, Nowoursynowska Street 159, 02-776 Warsaw, Poland; edmund_koziel@sggw.pl; 2 Department of Biological Sciences, Northern Illinois University, DeKalb, IL 60115, USA; jbujarski@niu.edu; 3 Institute of Bioorganic Chemistry, Polish Academy of Sciences, Noskowskiego 12/14, 61-704 Poznań, Poland

**Keywords:** viral intercellular transport, immunolocalization, plasmodesmata, 3D protein structure, *Prune dwarf virus*

## Abstract

*Prune dwarf virus* (PDV) is an important viral pathogen of plum, sweet cherry, peach, and many herbaceous test plants. Although PDV has been intensively investigated, mainly in the context of phylogenetic relationship of its genes and proteins, many gaps exist in our knowledge about the mechanism of intercellular transport of this virus. The aim of this work was to investigate alterations in cellular organelles and the cell-to-cell transport of PDV in *Cucumis sativus* cv. Polan at ultrastructural level. To analyze the role of viral proteins in local transport, double-immunogold assays were applied to localize PDV coat protein (CP) and movement protein (MP). We observe structural changes in chloroplasts, mitochondria, and cellular membranes. We prove that PDV is transported as viral particles via MP-generated tubular structures through plasmodesmata. Moreover, the computer-run 3D modeling reveals structural resemblances between MPs of PDV and of *Alfalfa mosaic virus* (AMV), implying similarities of transport mechanisms for both viruses.

## 1. Introduction

The *Bromoviridae* are a large family of plant RNA viruses which include many economically important pathogens of monocots and dicots [1]. The family contains 6 genera, with one of most interesting being the genus *Ilarvirus*. Amongst the *Ilarviruses*, an important species is *Prune dwarf virus* (PDV) [2,3]. This viral pathogen infects a whole range of hosts from plum, sweet cherry, and peach to various test plants [4,5]. The genome of PDV, like other *Ilarviruses*, is multipartite and consists of 3 viral RNA (vRNA) molecules: RNA1, RNA2 and RNA3 [1,6]. RNA1 and RNA2 encode two replication proteins, the “replicase” (P1 protein) and RNA-dependent RNA polymerase (RdRp, P2 protein), respectively [7,8]. Both P1 and P2 are required for replication of vRNAs and build replication complexes during viral infection [1,9]. RNA3 encodes two proteins, the movement protein (MP) and coat protein (CP) [6]. PDV-MP is involved in cell-to-cell transport [5,6]. Apart from forming the viral capsid, PDV-CP is needed for genome activation, and possibly also for cell-to-cell transport [5,6,10]. Our previous microscopic and bioinformatics studies showed that PDV infection induces changes in cell membranes and its cell-to-cell transport, likely similar to *Alfalfa mosaic virus* (AMV) [11,12]. This study focuses on sequential ultrastructural changes in cell organelles and PDV-induced structures in cucumber plants. Moreover, the study shows the potential mechanism of PDV intercellular transport based on both microscopic and bioinformatic analyses. Our observations reveal changes in chloroplasts, mitochondria and endomembranes, as well as in the cell wall and plasmodesmata, the latter carrying tubular structures for virus translocation.

## 2. Results

### 2.1. Symptomatology Induced by Prune Dwarf Virus (PDV) in Cucumis sativus

**Figure 1:**
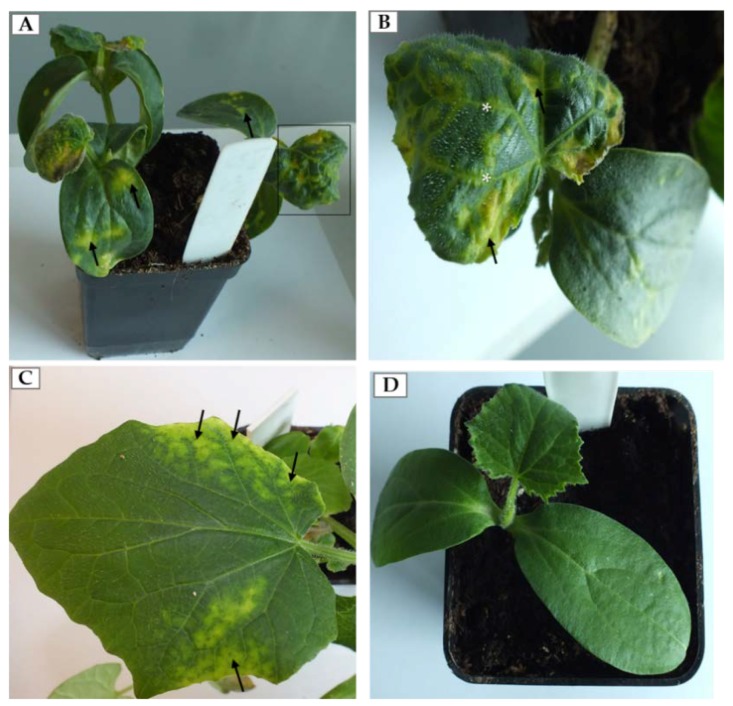
Morphological alternations of infection caused by *Prune dwarf virus* (PDV) on cucumber. (**A**) Chlorotic spots (arrow) on the cotyledons and leaves of cucumber infected by PDV (14 dpi). The black frame area is enlarged in (**B**); (**B**) Enlarged deformed leaf with chlorotic spots (arrow) and yellowing veins (*); (**C**) Yellowing of leaf blades along the edges (arrow) at 14 dpi; (**D**) Mock-inoculated cucumber plants without symptoms (14 dpi).

The first morphological changes induced by PDV were observed approximately seven days post-inoculation (dpi). At this stage, we observed chlorotic spots on the inoculated cotyledons (Figure 1A). From 14 dpi we also observed characteristic alterations of upper leaves (black frame on Figure 1A). From this point, the viral infection reached systemic leaves. Frequently, leaves of infected cucumber were deformed, chlorotic with yellowing veins (Figure 1B). On the other hand on some leaves, alterations at 14 dpi were slightly different, without deformations but instead with yellowing leaf blades along the edges (Figure 1C). Mock-inoculated plants did not develop symptoms on cotyledons and the leaves (Figure 1D).

### 2.2. Immunofluorescence Localization of Coat Protein (CP) in PDV-Infected Cucumber Leaves

**Figure 2:**
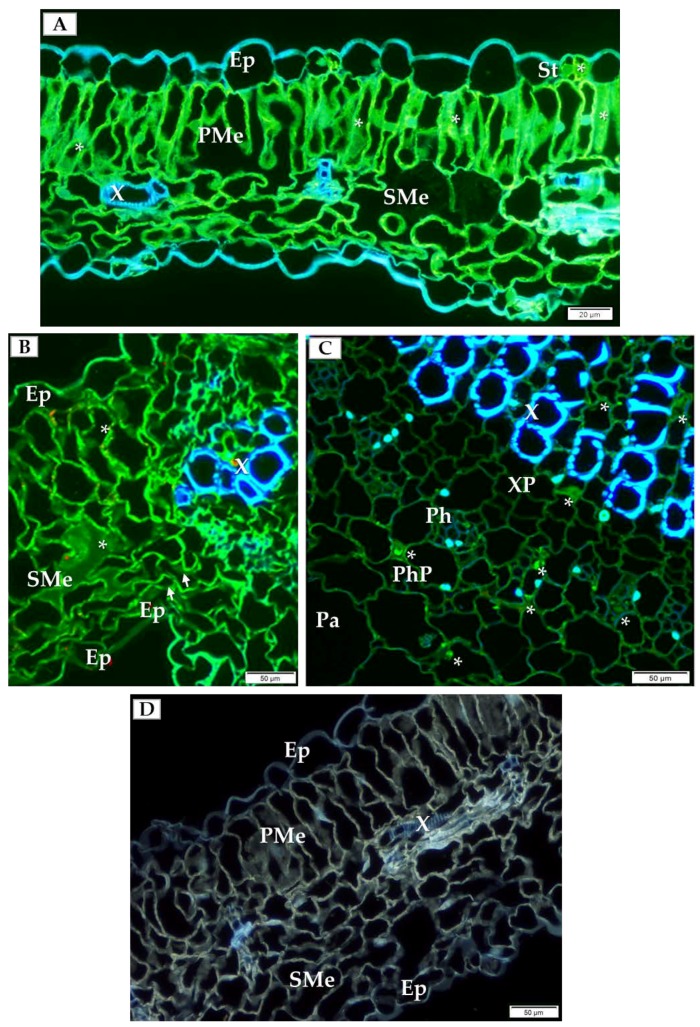
Immunofluorescence localization of PDV-coat protein (PDV-CP) epitopes in the infected cucumber leaf tissue of *Polan* variety (14 dpi). (**A**) Localization of the epitopes of PDV-CP (green fluorescence, marked with *) in stomata and palisade mesophyll cells (14 dpi), Bar 20 µm; (**B**) PDV-CP epitope (*) inside spongy mesophyll (14 dpi). Deformations of cell wall in spongy mesophyll cell are marked with white arrows, Bar 50 µm; (**C**) Cross section of cucumber leaf through main vein with presence of CP (*) in xylem and phloem parenchyma (14 dpi), Bar 50 µm; (**D**) Section of mock-inoculated leaf without PDV-CP, Bar 50 µm. Abbreviations: Ep—epidermis, St—stomata, PMe—palisade mesophyll, SMe—spongy mesophyll, Pa—parenchyma, X—tracheal element, XP—xylem parenchyma, Ph—phloem, PhP—phloem parenchyma.

In order to choose which cell types to analyze by detailed TEM (transmission electron microscope) and double-immunogold labeling, we initially performed immunofluorescence labeling of PDV-CP to localize viral particles inside specific cells and types of tissue inside the cucumber leaf. At 14 dpi, PDV-CP was localized in various tissues with different frequency in the individual leaf parts. Most commonly, the occurrence of CP was observed in the stomata, palisade and spongy mesophyll cells (Figure 2A,B). Moreover, in the infected leaves, the deformations were visible in spongy mesophyll cells (Figure 2B). Presence of CP was also confirmed in the central vascular bundle in the main vein, confirming systemic infection (Figure 2C). The presence of PDV-CP was noticed in both xylem and phloem parenchyma (Figure 2C). No deformations and PDV-CP localization are shown (Figure 2D).

### 2.3. Ultrastructural Alterations in Cucumber Cells During PDV Infection

**Figure 3:**
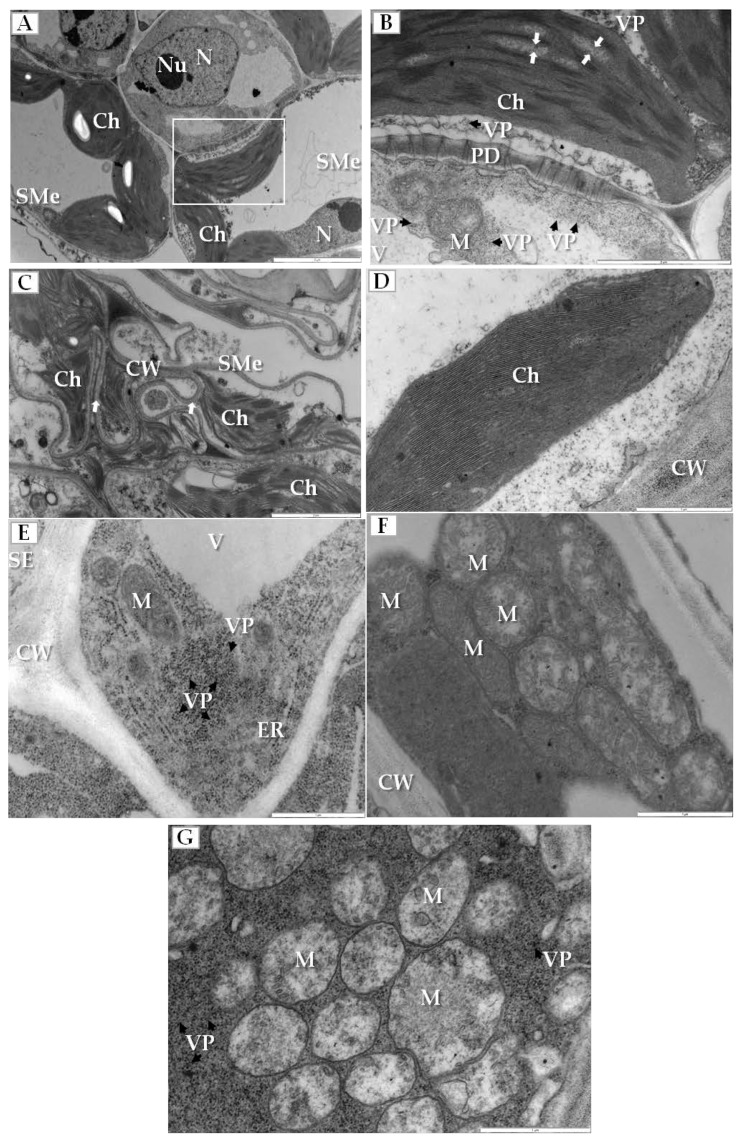
Ultrastructural alterations in cucumber organelles and cell wall during PDV infection. (**A**) Section through the PDV-infected leaf blade, with spongy mesophyll cells with changed chloroplasts. The white framed area is enlarged in (**B**), Bar 5 µm; (**B**) Spongy mesophyll cell from (**A**) with electron translucent regions in the stroma between thylakoids (white arrow) and also with viral particles present (VP, black arrow), Bar 2 µm; (**C**) Altered spongy mesophyll cell from infected cucumber with disintegrated chloroplasts and the cell wall irregular and deformed (white arrow), Bar 2 µm; (**D**) Parenchyma cell of from mock-inoculated cucumber without altered chloroplast and cell wall, Bar 1 µm; (**E**) Companion cell from infected cucumber with enlarged endoplasmic reticulum cisterns and the PDV particles present in cytoplasm (VP, black arrow), Bar 1 µm; (**F**) Fragment of a companion cell from infected cucumber showing mitochondria with reduced cristae and large electron translucent regions- early stage of mitochondrial changes, Bar 1 µm; (**G**) Region in the cytoplasm of a companion cell filled with PDV particles (VP, black arrow), and with no cristae in the mitochondria, Bar 1 µm. Abbreviations: SMe—spongy mesophyll, SE—sieve tube, Ch—chloroplast, N—nucleus; Nu—nucleolus, PD—plasmodesmata, V—vacuole, M—mitochondrion, CW—cell wall, ER—endoplasmic reticulum, VP, black arrow—viral particles.

Sequential changes in the structure of some crucial plant cell organelles were found in the infected cucumber leaves. Most significantly, the electron translucent regions appeared in the stroma between thylakoids in chloroplasts of spongy mesophyll cells (Figure 3A,B). Moreover, the outer membranes of disintegrated chloroplasts and deformed thylakoids were observed in the cytoplasm of these cells that often also had irregular and deformed cell walls (Figure 3C). Such changes were not observed in chloroplasts from mock-inoculated cucumber plants (Figure 3D). On the other hand, altered mitochondria were mainly observed inside companion cells of the phloem; these cells contained many viral particles and showed the enlarged endoplasmic reticulum (ER) cisterns (Figure 3E) and different stages of other mitochondrial alterations (Figure 3F,G). Many mitochondria had reduced cristae and large electron translucent regions within the mitochondrial matrix (Figure 3F). When the companion cell was filled with PDV particles the mitochondrial cristae completely disappeared (Figure 3G).

**Figure 4:**
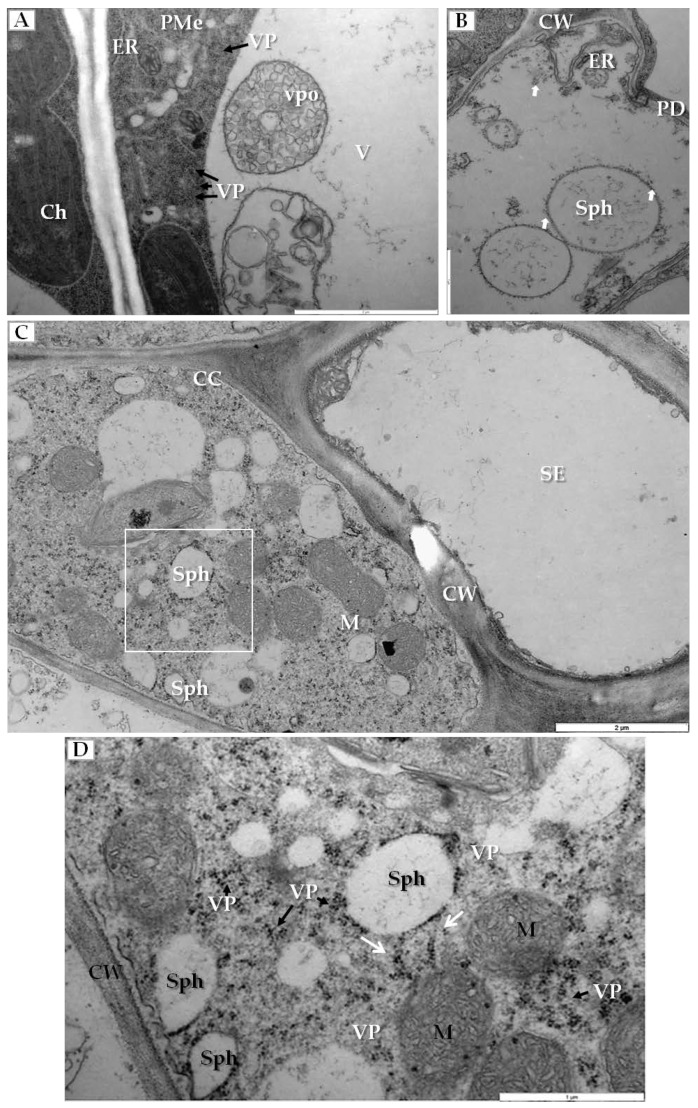
Membranous structures induced by PDV infection. (**A**) Fragment of palisade mesophyll cell from PDV infected cucumber with viral particles (VP, black arrow) in cytoplasm and vesicle pockets inside vacuole, Bar 2 µm; (**B**) Mesophyll cell with spherules carrying viral particles on the surface (white arrow), Bar 1 µm; (**C**) Section through a phloem cell with companion cell and sieve tube, showing various numbers of membranous structures and spherules. The white frame area is enlarged in (**D**), Bar 2 µm; (**D**) A fragment of companion cell with viral particles present, arranged in line (white arrow) connected to the spherule, Viral particles in cytoplasm (black arrow) Bar 1 µm. Abbreviations: PMe—palisade mesophyll, SE—sieve tube, Ch—chloroplast, PD—plasmodesmata, vpo—vesicle pockets, Sph—spherules V—vacuole, M—mitochondrion, CW—cell wall, ER—endoplasmic reticulum, VP, black arrow—viral particles.

Another type of cellular changes was associated with the generation of membranous structures in infected cells. In palisade mesophyll cells that contained viral particles, the enlarged ER and vesicle pockets were visible inside the vacuoles (Figure 4A). Simultaneously in mesophyll cells, we observed spherules (vesicles with viral particles on their surface) (Figure 4B). Similar changes were noticed inside companion cells (Figure 4C), where many spherules and viral particles were found (Figure 4D).

**Figure 5:**
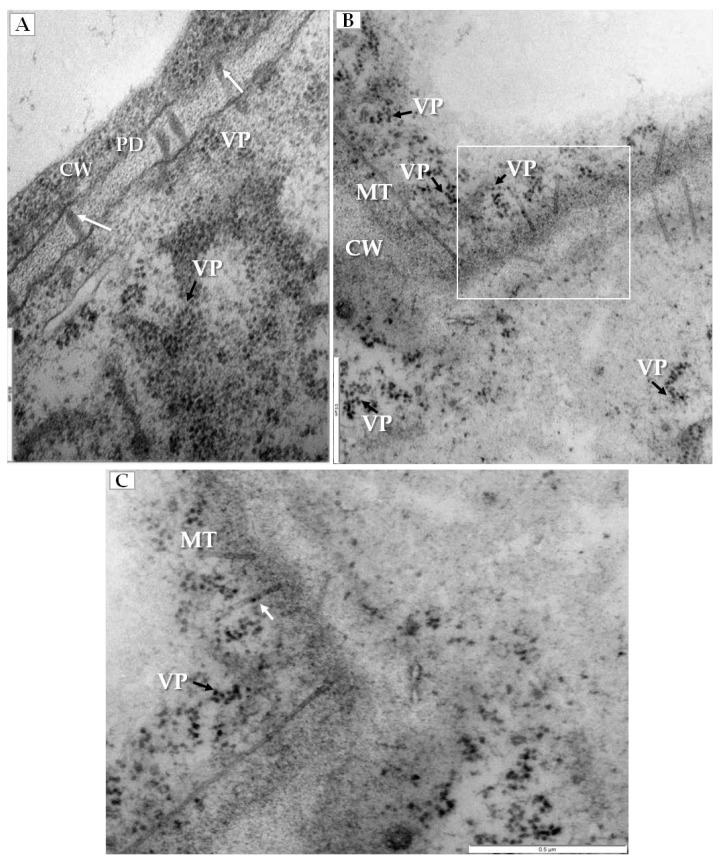
Changes in plasmodesmata in PDV-infected cucumber leaf. (**A**) Section of mesophyll cell with extended plasmodesmata and viral particles (VP, black arrow). Viral particles present inside plasmodesmata and indicated with a white arrow, Bar 0.5 µm; (**B**) Fragment of mesophyll cell containing viral particles (VP, black arrow) near tubular structures induced by PDV-MP. The white frame area is presented in (**C**), Bar 0.5 µm; (**C**) This enlarged fragment of infected mesophyll cell shows viral particles inside tubular structures induced by PDV MP (white arrow). Viral particles (VP) in cytoplasm near plasmodesmata are marked with black arrow, Bar 0.5 µm. Abbreviations: CW—cell wall, PD—plasmodesmata, MT—movement protein induced tubular structures, VP, black arrow—viral particles.

PDV infection also affected the plasmodesmata, especially in mesophyll cells that had PDV particles (Figure 5A). Namely, the plasmodesmata kept the tubular structures that passed through cell wall (Figure 5B), and the tubular structures frequently carried the intact PDV particles (Figure 5C).

**Figure 6:**
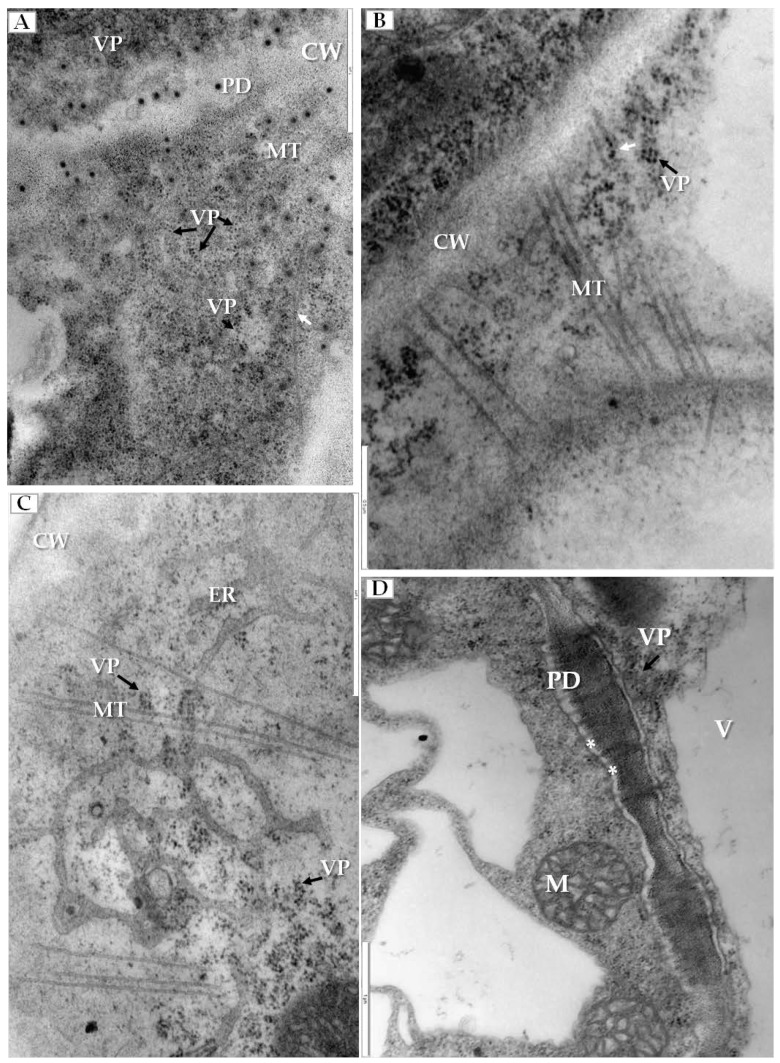
Tubular structures and changes in secondary plasmodesmata in PDV infected cucumber plants. (**A**) Phloem parenchyma cell with transverse sections of plasmodesmata. Viral particles are visible in cytoplasm (VP, black arrow) and in tubular structures directed to plasmodesmata (white arrow), Bar 1 µm; (**B**) Tubular structures with viral particles inside (white arrow). Tubular structures pass through cell walls of three cells. Viral particles (VP, black arrow) near plasmodesmata, Bar 0.5 µm; (**C**) PDV particles (VP, black arrow), tubular structures and enlarged endoplasmic reticulum cisterns in phloem parenchyma cell, Bar 1 µm; (**D**) Viral particles (VP, black arrow) near secondary plasmodesmata in phloem parenchyma cell. Moved plasmalemma (*) near secondary plamodesmata, Bar 1 µm. Abbreviations: CW—cell wall, PD—plasmodesmata, ER—endoplasmic reticulum, MT—movement protein induced tubular structures, VP, black arrow—viral particles, M—mitochondrion; V—vacuole.

**Figure 7:**
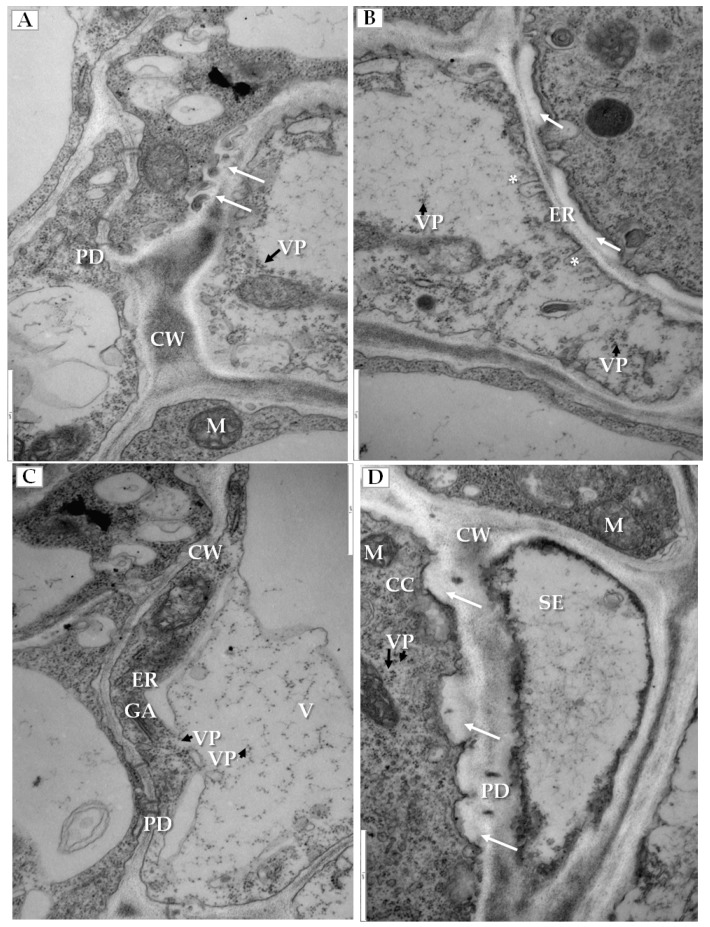
Changes in cell wall structure and plasmolemma in phloem cells of PDV infected cucumber leaves. (**A**) Fragment of phloem parenchyma with cell-wall invaginations (white arrow). Viral particles (VP, black arrow) in phloem parenchyma cell, Bar 1 µm; (**B**) Fragments of phloem parenchyma cells with irregular cell wall (white arrow) and plasmalemma invaginations (*) and with viral particles (VP, black arrow), Bar 1 µm; (**C**) Presence of viral particles (VP, black arrow) inside the vacuole with the vacuolar lumen having connections to the cytoplasm of parenchyma cell, Bar 1 µm; (**D**) An irregular and thicker cell wall (white arrow) between companion cell with viral particles (VP, black arrow) and sieve tube, Bar 1 µm. Abbreviations: CW—cell wall, PD—plasmodesmata, ER—endoplasmic reticulum, GA—Golgi apparatus, MT—movement protein induced tubular structures, VP, black arrow—viral particles, SE—sieve tube, CC—companion cell, M—mitochondrion, V—vacuole.

We have also clearly confirmed the connections between tubular structures and viral particles in transverse sections of plasmodesmata (Figure 6A). In many cases, viral particles could be observed inside tubules which pass through plasmodesmata of more than two cells (Figure 6B). Frequently near the cell wall, the enlarged ER could be observed (Figure 6C). Viral particles were found near the secondary plasmodesmata (Figure 6D). Changes in the plasmodesmata structure paralleled alterations in the cell wall, especially in the phloem parenchyma that had many cell wall invaginations (arrow, Figure 7A) or irregular structures (Figure 7B). Such irregularities were not observed in uninfected cells. In case of cells with irregular cell walls, the plasmalemma was often invaginating in many regions (Figure 7B). In some cells with irregular wall structures, we observed the presence of viral particles inside the vacuole (Figure 7C), with the vacuolar lumen having connections to cytoplasm (Figure 7C). Alterations near the cell wall were also observed in the companion cells and sieve tubes. The cell wall around plasmodesmata was thicker whereas in companion cells numerous viral particles were observed (Figure 7D).

### 2.4. Modeling of 3D Structures of PDV-0599 and Alfalfa Mosaic Virus-VRU (AMV-VRU) Movement Proteins (MPs)

**Figure 8:**
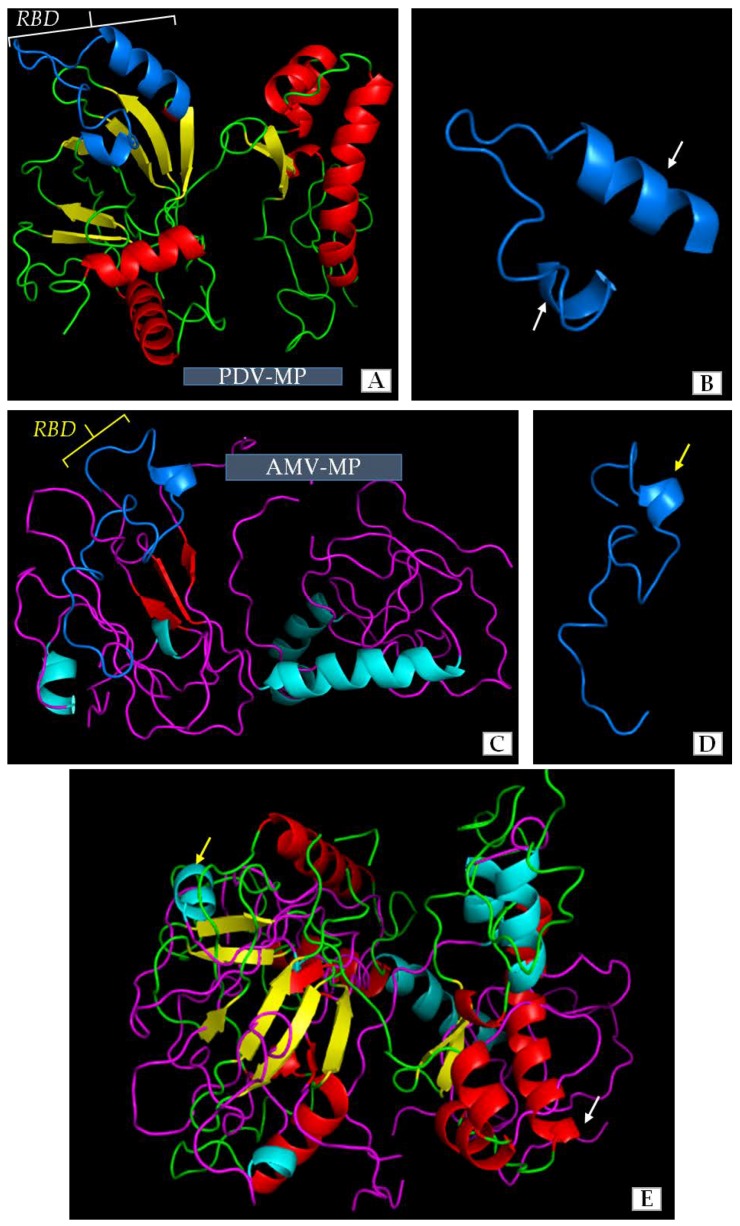
Three-dimensional (3D) model structures of the PDV movement protein (PDV-MP) and AMV movement protein (AMV-MP). (**A**) 3D model of PDV-MP structure. The colors show the particular elements of the secondary structure, as follows. Green indicates the fragments of straight polypeptide chain, red—α-helical fragments, yellow—β-card fragments, blue-RNA binding domain (RBD); (**B**) The blue color represents the RBD structure of PDV-MP, enlarged from (**A**). White arrows indicate two α-helical fragments of this domain; (**C**) 3D model of AMV-MP structure. The colors show the particular elements of the secondary structure as follows. Purple indicates the fragments of straight polypeptide chain, green—α-helical fragments, red—β-card fragments, blue—RNA binding domain (RBD); (**D**) The blue color show RBD structure of AMV-MP enlarged from (**C**). Yellow arrow specifies the α-helical fragment of this domain; (**E**) Overlaid of 3D models of PDV-MP and AMV-MP. Yellow and white arrows show AMV-MP and PDV-MP, respectively. The colors represent the particular elements as shown in (**A**,**C**) for PDV-MP and AMV-MP, respectively.

Our previous studies and literature data revealed some similarities among the amino-acids of movement protein (MP) of PDV and that of *Alfalfa mosaic virus* (AMV). AMV has a well-described mechanism of cell-to-cell transport or the role of its MP in viral infection. However, the structure of PDV-MP is unknown, whereas the function of MPs amino acids sequence is only one side of coin. The 3D structure of viral MPs in general has a significant influence on function during viral infection and especially on cell-to-cell transport. We compared the structures of MPs of PDV and AMV to clarify similarities between both viruses to better understand their function. The use of Jalview and AIDA server enabled us to distinguish regions with characteristic structure. Apparently, most of the sequence in both MPs formed a simple loop structure. There were different numbers of α-helical and β-sheet regions in both structures (Figure 8A–D). The MP of PDV-0599 had seven α-helical (5 short helices-1-3 coils, and 2 longer helices with 5 coils) and eight β-sheet regions (Figure 8A). RBD localized between 56–85 aa in PDV-MP sequence consisted of one loop and two α-helices. AMV-MP had less number of complex regions (Figure 8) with five α-helices (4 short helices-1-3 coils and one long helix with 4 coils) and three β-sheets. These regions were localized at equivalent points within the overall protein structure (Figure 8A,C). RBD was also identified between 56–85 aa in AMV-MP, being quite similar to RBD of PDV, except that AMV had only one helical region (Figure 8D). Despite the differences in number of α-helical and β-sheet regions, an overlay of the 3D structures indicated more similarities than differences between both proteins (Figure 8E).

### 2.5. Localization and Quantification of PDV-CP and MP in Infected Cucumber Leaves by Double-Immunogold

**Figure 9:**
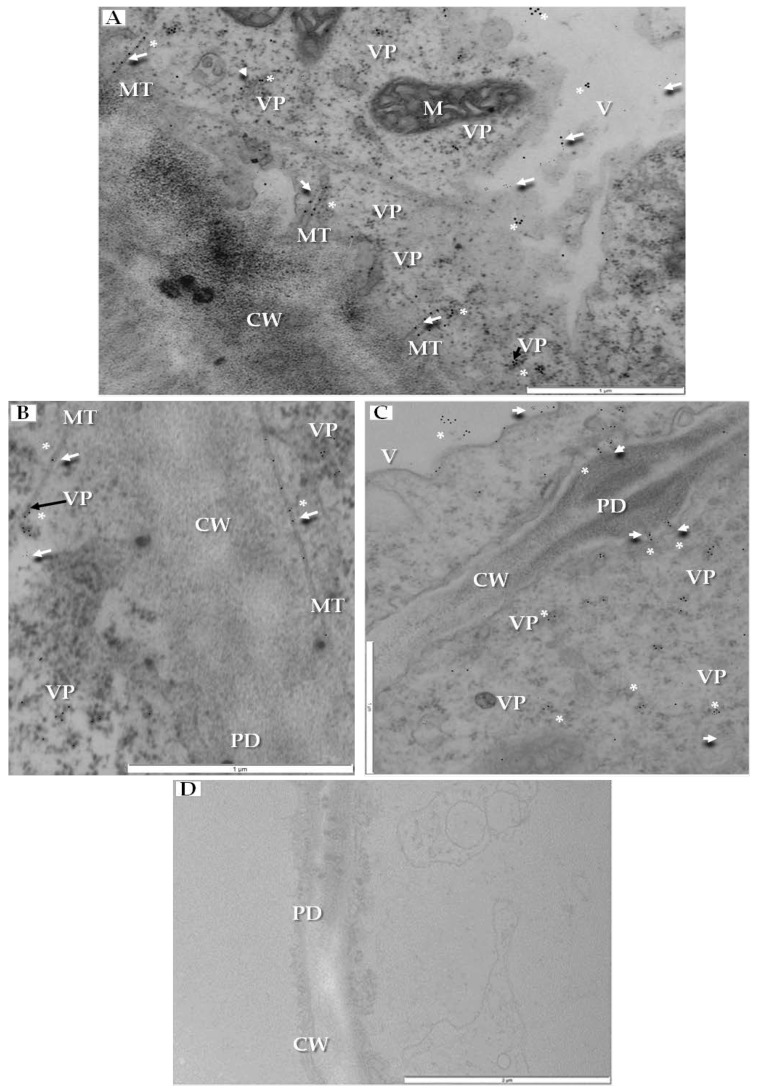
Double-immunogold localization of PDV movement protein (MP) and coat protein (CP) in infected cucumber leaf tissues (14 dpi). (**A**) MP epitopes (white arrow) and CP epitopes (*) colocalized in tubular structures in plasmodesmata, cytoplasm and vacuole. CP epitopes (*) on the surface of viral particles. Labeled viral particles in cytoplasm (VP, black arrow), Bar 1 µm; (**B**) Viral particles (VP, black arrow) in tubular structure. Co-localization of MP epitopes (white arrow) and CP epitopes (*) are visible, Bar 1 µm; (**C**) Two types of gold particles (* and white arrow) from CP and MP co-localized in cytoplasm and in plasmodesma, Bar 1 µm; (**D**) Fragment of parenchyma cell from mock-inoculated cucumber without MP and CP epitopes, Bar 1 µm. Abbreviations: CW—cell wall, PD—plasmodesmata, M—mitochondrion, V—vacuole, MT—movement protein induced tubular structures, VP—viral particles.

Local (cell-to-cell) transport could be studied by localizing two major viral proteins, CP and MP in tubular structures that contain virus particles, by using double-immunogold technique. We have co-localized CP and MP in specific parts of infected cell. Both epitopes were detected together inside the tubular structures inside plasmodesmata (Figure 9A), but CP epitope was located on viral particles not only in tubular structures but also in the cytoplasm (Figure 9A,B). Moreover, MP and CP co-localized inside secondary plasmodesmata and in the cytoplasm near plasmodesmata (Figure 9C). No PDV-CP and MP epitopes were detected in mock-inoculated cucumber plants (Figure 9D).

To quantify the co-localization of PDV-CP and MP, the statistical dual labelling was performed. The values of the odds ratio (OR) and the results from Fisher’s test for double-immunogold parameters (estimated statistical values *p* < 0.001) revealed that the zero hypothesis—i.e., that localizations of CP and MP are independent of each other—has to be rejected. Positive values of the OR in case of plasmodesmata containing tubular structures (OR = 12.04), cytoplasm (OR = 5.66), and vacuole (OR = 2.40), with a high incidence of double positives coupled with a high incidence of double negatives, indicated the statistically significant co-localization of MP and CP in these cellular compartments (Table 1). Moreover, differences between the OR values showed that the strongest co-localization of PDV-CP and MP was observed in plasmodesmata and in tubular structures. The co-localization level was lower in the cytoplasm and the lowest in the vacuole.

## 3. Discussion

In this work we conducted ultrastructural analyzes of PDV-infected tissues in order to address the mechanisms of pathogenesis and virus transportation in cucumber. The results of these studies allowed us to draw several important conclusions. The first group of results regards the morphological changes and symptoms of PDV infection. PDV is present all over the world [1] causing increasing problems in a wide range of plant hosts [3,13], especially in orchard trees from the genus *Prunus* (sweet or sour cherry, plum or peach) [12]. PDV is well propagated by mechanic transmission [14,15,16], but less importantly by pollen and seeds [8,13,17], and interferes with the vegetative propagation of trees [14,15,16].

Different strains/isolates of PDV can generate different symptoms in the same host [3,12], causing different plant diseases [7]. Among the effects caused by all PDV strains [3,8], there are dwarfing and reduction in flowers and fruits [13]. Two phases of disease [4] include a “shock” phase observed during first two years [8] followed by the asymptomatic chronic phase [3,8]. During “shock” phase [8,18,19], not only does PDV induces strain-specific chlorotic spots/rings and mottle or line patterns on leaf blades [20,21,22], but also reduces the growth of shoots [23] or the number of flowers [5]. The flowers remain strongly deformed, without stamens [8,23]. The chronic phase involves growth reduction of plants whereas the concentration of the virus is generally very low, barely detectable by DAS-ELISA [24], best detected between March and May [24]. The PDV disease can easily be overlooked in nurseries [3,13]. Therefore, the presence of PDV is verified on test plants including *Cucumis sativus* (especially cv. Wisconsin and cv. Polan), *Cucurbita maxima* (for example cv. Buttercup) and *Nicotiana tabaccum* cv. Samsun [3,4,20,24].

In our experiments, PDV-0599 induced local chlorotic spots on the inoculated cotyledons of *Cucumis sativus* cv. Polan 7 dpi, similar to those presented by Fulton [20]. Moreover, further alteration/deformation and chlorotic lesions of fully developed leaves were observed 14 dpi, as an element of systemic spread, also described by Fulton [20]. Leaf deformations and chlorotic lesions were documented on plums [3,8] or *Nicotiana tabaccum* cv. Samsun. Kozieł [25] noted chlorotic alternations and deformation of the upper side of systemic leaves after 15 dpi, also observed by Waterworth and Fulton [26]. Other members of the *Bromoviridae* family, such as AMV can induce leaf vein necrosis in tobacco leaves, often reaching vascular tissues [27,28,29].

Cotyledons and fully developed leaves of cucumber have different morphology, with cotyledons retaining some embryonic features in anatomical structure, whereas upper leaves are characteristic for mature plants [30]. The structure of fully developed cucumber leaves is more similar to those of *Prunus* sp. [30]. Therefore, we have used systemically infected cucumber leaves as models for ultrastructural investigations of the effects of PDV in mature plant organs.

Secondly, by using immunofluorescence we localized the PDV-CP epitopes in the cucumber tissue. In general, the PDV-CP can reach much higher levels during viral infection than other PDV proteins [5,31]. Apart from forming viral particles, the CP of *Illarviruses* is engaged in the asymmetric (+)/(−) strand RNA synthesis, translation of viral RNA, and during both intercellular and systemic transport [10]. Previously, using the immunofluorescence and TEM, Kozieł et al. [32] co-localized PDV-CP and the replicase P1 protein, as an efficient marker of ongoing PDV replication. The presence of PDV-CP epitopes in palisade and spongy mesophyll cells at 14 dpi confirmed the virus spread to various tissues of cucumber, which corresponded to deformations in mesophyll cells, also observed in tobacco [32]. However, in contrast to tobacco, we did not observe necrosis in cucumber vascular bundles at 14 dpi, likely reflecting varying reactions of tobacco and cucumber to PDV.

The systemic transport of PDV seems to occur equally in sieve tubes and xylem vessels, as shown by us in tobacco [33]. Similar results were observed in natural hosts. Malinowski and Zawadzka [24] showed that the best materials for PDV diagnostic tests by Double Antibody Sandwich Enzyme-Linked Immunosorbent Assay (DAS-ELISA) were vascular bundles of leaves and shoots, also true for other *Bromoviridae*. AMV-CP localized inside the palisade and spongy mesophylls but not in vascular tissues at 7 dpi in *Nicotiana benthamiana* [34,35,36] but at 14 dpi the AMV-CP epitopes were located in mesophyll as well as in vascular tissues. The comparisons of CP sequences of PDV and AMV revealed high similarity at selected regions [25], suggesting similarities between infection mechanisms for both viruses.

Other observations of this work concern ultrastructural changes in cucumber cells. One group of ultrastructural changes induced by plant viruses can be classified as directly connected with viral genome replication and/or with viral particle assembly [37,38]. Different membranous structures can form vesicles with single or double membranes, tubules or stacked membranes [38]. Yet the more complex changes are related to plant reactions against infection, such as those associated with the presence of R (resistance) genes and the accumulation of levels of reactive oxygen species (ROS) [39]. In resistant plants, ROS is frequently associated with the hypersensitive response (HR) [40]. Regardless of resistance or susceptibility, ROS often alters mitochondria and chloroplasts. ROS damage of chloroplasts is reflected by chlorotic lesions or leaf discoloration [37,41]. Plant viruses change the expression of plant genes leading to structural deformations. Plasmodesmata (PD) and the cell wall are amongst the highly modified structures [42,43]. For PDs, the host plant tries to block the path to stop the viral cell-to-cell transport [31], e.g., via callose depositions [44]. *Tobacco mosaic virus* (TMV) is known to induce the synthesis of β-1,3-glucanases which degrade callose, preventing the PD blockage [45]. Cell wall changes are not fully explored in plant-virus interactions [41].

Previously, we reported the localization of sites of PDV replication in tobacco [32]. Here, we characterize for the first time the ultrastructural alterations in PDV-infected cucumber. Three major groups of cellular alterations can be distinguished. The first group involves sequential changes in chloroplasts, mitochondria and ER. In early stages, the observed changes did not parallel the presence of PDV particles, with the electron translucent regions in chloroplasts, reduced cristae in mitochondria and the enlarged ER cisterns. Later, the viral particles were observed while both chloroplasts and mitochondria were more severely damaged, likely because of ROS. ROS is known to induce programmed cell death [37]. Favali and Conti [46] observed electron translucent regions in chloroplasts of bean infected with AMV. Whereas the enlargement of ER is probably due to the high activity in protein synthesis, such as P1 and P2 replicase proteins, virion assembly (CP) or cell-to-cell transport (MP). In fact, our data on the immunogold localization in tobacco showed PDV-CP frequently localizing to the ER [32], also reported for BMV in *N. benthamiana* [9,47]. Moreover, Kozieł [25] observed severe damage to chloroplasts and mitochondria in PDV-infected tobacco. Apart from ROS, some changes may result directly from accumulation of viral particles, as shown for AMV accumulating in chloroplasts [46,48,49].

The second group of alterations concerned membranous structures that can serve as markers of viral genome replication [9,50]. In cucumber infected by PDV vesicular spherules were inside the vacuoles of mesophyll and phloem cells, similar to P1 protein in tonoplast of tobacco [32], These spherules serve as ultrastructural evidence for ongoing PDV RNA replication; for BMV such vesicles differentiate from the ER membranes [9,12,47]. Bamunusinghe et al. [51] demonstrated that BMV-induced spherules depend on the BMV CP, with different CP mutations blocking the formation of viral particles whereas other mutations blocked the formation of membranous structures [51]. The AMV replication occurs at tonoplast with P1 and P2 proteins and the AMV RNAs co-localizing [52]. Thus, membrane alterations caused by PDV are more similar to AMV than BMV, both associating with the vacuole. The association of CP with membrane alterations needs further studies for both BMV and PDV; the preliminary data from immunogold localized the PDV epitopes in the vacuole and at the tonoplast [12,32].

The third group of changes included alterations of the cell wall and plasmodesmata. The cell wall was modified mainly in phloem parenchyma with many invaginations and an irregular wall structure. These have also been observed for other plant viruses. For instance, Otulak-Kozieł et al. [41] revealed a higher level of pathogenesis-related protein 2 (PR-2) during a compatible infection by necrotic strain of *Potato virus Y* (PVY^NTN^). The hydroxyproline-rich glycoproteins (HRGP) (extensin) were induced, whereas the cellulose synthase catalytic subunit (CesA4) was downregulated.

Proteins for cell wall metabolism are important because they affect the spread of the virus. Analysis of the deposition of CesA4, PR-2 and HRGP within the apoplast and symplast verified their trafficking in potato cell wall in response to PVY^NTN^ [41]. For BMV, AMV and *Cowpea chlorotic mottle virus* (CCMV), changes in the size exclusion limit (SEL) of plasmodesmata were reported [53,54], causing considerable increase of the SEL so that the viral transport can occur [53]. Here we confirm the presence of tubular structures containing PDV particles inside plasmodesmata in cucumber, often observed for plant viruses [37]. Similar tubular structures containing viral particles, protruding from the cell surface were reported for protoplasts infected with BMV or AMV [55,56,57,58,59,60]. Interestingly, the MPs of BMV or AMV were the only proteins needed for tubule induction in protoplasts. We conclude that ultrastructural modifications of plasmodesmata caused by PDV are similar to those of AMV and BMV.

The computer overlay of the 3D models showed some level of similarity between 3D structures of PDV-0599 MP and AMV-VRU MP, as a whole and specifically between the RNA binding domains (RBD). Both MPs were shown to be the most related within the *Bromoviridae* family [11]. Along these lines, Codoñer et al. [61,62] stated that PDV, *Prunus necrotic ringspot virus* (PNRSV) and AMV are phylogenetically related. Moreover, the MPs of PNRSV, BMV or even *Cucumber mosaic virus* (CMV) could replace the function of MP during AMV infection [63,64], implying similar mechanisms of cell to cell transport [3,11]. Numerous contact points have been revealed between 3D structures of MP and CP in PNRSV [62], suggesting that both proteins may interact during infection.

Finally, by using the double-immunogold co-localization we observed strong correlation between PDV CP and MP in the context of cell-to-cell movement. Plant viruses can move cell-to-cell either as RNA-protein complexes (RNP complex) [63,64,65] or as the entire viral particles. The first mechanism could be either dependent or independent of CP. The CP independent transport is characteristic for CCMV where CCMV-MP forms a complex with viral RNA while the unbound MP increases the SEL of plasmodesmata [53,66]. With the second mechanism both MP and CP are needed for cell-to-cell transport [67], e.g., for CMV [68], where CMV-MP increases the SEL [69,70,71,72] but the virus is transported as an RNP complex consisting of vRNA, MP and CP; the latter stabilizes the complex during delivery to PD [73,74]. The C-terminal basic arm of CP is probably involved in these interactions [68].

When the entire virions are transported, e.g., in the case of AMV [56] or BMV [55,57], both MP and CP are needed for intercellular transfer [75], with the MPs forming tubular structures [56,57] whereas CP on the surface of virions interacts with MP [9]. Our results with PDV showed strong double-immunogold co-localization of CP and MP within the tubular structures of PD, implying the mechanisms comparable to those of AMV and BMV [9,55,56]. However, both the computer modeling of MP and the MP-CP co-localization suggest PDV transport to be more closely related to AMV than to BMV. Whereas the co-localization of PDV-CP and PDV-MP near tonoplast indicates that vacuole is engaged during interaction of both proteins.

## 4. Materials and Methods

### 4.1. Virus Inoculation

To investigate PDV induced pathogenesis, cell-to-cell transport, and immunolocalizations, the test plant *Cucumis sativus* cv. Polan was selected [3,13], as a cultivar very susceptible for a broad range of PDV isolates/strains. The seedlings were mechanically inoculated at a two cotyledon stage (7 days old) with the PDV-0599 suspension in 0.01 M potassium-phosphate buffer containing diethyldithiocarbamic acid (DIECA) [32]. The inoculum was prepared from plum-infected flower buds, obtained from the Institute of Horticulture in Skierniewice, Poland.

### 4.2. Plant Material Preparations for Immunofluorescence and Immunofluorescence Localization of Coat Protein (CP) in Cucumber Leaves

14 days after PDV-0599 infection, fragments of cucumber leaf blades were fixed in paraformaldehyde and embedded butyl-methyl- acrylate resin (BMM). Parameters of fixation and embedding were as previously described by Kozieł et al. [32].

To investigate the presence of PDV in various plant host cells, immunofluorescence localizations of PDV-CP were performed. Cucumber leafs embedded in BMM were cut into 3 µm thick section. Sections were placed on special Poly-l-Lysine slides (Thermo Scientific, Nettetal, Germany) in a drop of distilled water. Slides with sections were then placed for one day on a thermoblock warmed to 45 °C. This procedure attaches and immobilizes the sections on the slides [32]. In the next step slides with cucumber leaf sections were treated and incubated in a series of solutions from acetone (washing resin from section surface), distilled water, phosphate-buffered saline (PBS) (pre-incubation), PBS containing 2% bovine serum albumin (PBS-BSA) (for blocking of non-specific epitopes) and again with PBS (for washing off residual PBS-BSA). The above series of treatments and further dilution of and incubation with antibodies were performed according to the procedure described by Otulak et al. [76], with modification of Kozieł et al. [32]. PBS-washed slides were treated with primary purified rabbit polyclonal antibody anti-CP-PDV (Bioreba, Reinach, Switzerland). Four rinses in 0.01 M PBS buffer with 0.05% Tween 20, and then one rinse in 0.01 M PBS were applied. The slides were then treated with secondary anti-rabbit antibody IgG with attached AlexaFluor^®^488 (Jackson Immuno Research Europe Ltd., Cambridgeshire, UK) for 1 h in the dark. After incubation, the sections were washed by PBS-Tween and PBS and stained by DAPI (4′, 6-diamidino-2-phenylindole). Immuno-stained sections were imaged in a AX70 PROVIS fluorescent microscope with Olympus UP90 HD camera (Olympus, Warsaw, Poland). Images were acquired using Olympus Cell Sense Standard Software (Olympus, Center Valley, PA, USA, version 1.18).

### 4.3. Preparation of Plant Material for Transsmision Electron Microscopy (TEM) and Double-Immunogold Localization of CP and MP

14 days after PDV inoculation, fragments of leaf blades with symptoms of infection and mock-inoculated (healthy) plants were cut [76,77]. Samples of leaf blades were fixed and then embedded in EPOXY resin according to the procedure described in ref. [78], with modification in Kozieł et al. [32]. Then samples in EPOXY resin were cut into ultrathin sections (100 nm) and mounted on copper (for ultrastructural analyses) or nickel grids (for double-immunogold localization) [76,77,78].

### 4.4. Comparative Analyses of 3D Models of PDV-0599 and AMV-VRU

To investigate the potential role of PDV-0599 MP in plasmodesmata changes that we observed in cucumber leaf, we performed some bioinformatics analyses. From previously published data, we were aware that amino acid sequences of MP protein were most similar to sequence of Alfalfa mosaic virus-VRU (AMV-VRU, GeneBank: AAD04692.1) [11]. The amino acid sequences of MPs of PDV-0599 and AMV-VRU were taken from NCBI Protein database [79]. and the secondary structures were analyzed with Jalview Desktop 2.10.3b.1. (University of Dundee, Dundee, Scotland) [80]. The 3D structures of whole MPs for both viruses were predicted on AIDA Server (San Diego, CA, USA) [81], according Kozieł et al. [32] whereas the most important region of PDV and AMV MPs was compared based on RBD (RNA binding domain). To compare the whole 3D structures and RBDs, the number, type and dimensional localization of structural regions were analyzed. Visualization of MP 3D structure was prepared in PyMOL 2.1.0 (Cambrige, UK) [82].

### 4.5. Double-Immunogold Localization and Quantification of PDV-CP and MP in Cucumber Leaves

Double-immunogold localization of PDV-CP and MP was performed in addition to the single-immunofluorescent localization of CP. Two sets of antibodies were used. The first set consisted of primary rabbit polyclonal antibody anti-PDV-CP (Bioreba, Switzerland), which targets the whole spectrum of PDV strains, whereas the secondary polyclonal anti-rabbit antibody coupled with colloidal gold (20 nm gold particles) was from Sigma Aldrich, Warsaw, Poland. As the second set, a primary mouse polyclonal antibody PDV-MP (detecting fragment TKGKSSLENVKEAESVH of MP) (GeneCust, Ellange, Luxemburg) PDV-0599 (GeneBank: ADG65215.1) and secondary anti-mouse antibodies coupled to 10nm gold particles (Sigma Aldrich, Warsaw, Poland) were used. The embedded plant material (see above TEM section) was cut on an ultramicrotome to the 100 nm sections (100 nm, placed on nickel grids and treated, as described in refs. [32,77]. Then, the grids were washed in 0.01 M PBS-Tween 20 and PBS, incubated for one hour with the primary antibody rabbit anti-CP-PDV, at a dilution as in Kozieł et al. [32]. After incubation, grids were washed in PBS-Tween 20 followed by PBS. The grids were incubated with secondary anti-rabbit IgG conjugated with colloidal gold (20 nm) for 1 h, (dilution as in [32]). After this, incubation grids were washed with PBS-Tween 20 and PBS, incubated with primary mouse polyclonal antibody PDV-MP (at a 1:100 dilution) in PBS, washed in PBS-Tween 20 and PBS, and finally incubated with secondary anti-mouse antibodies associated with 10nm gold particles. Lastly, the grids were rinsed, stained with uranyl acetate and observed in transmission electron microscopy [78]. Double-immunogold colocalization of CP and MP was analyzed using a 2 × 2 contingency table as described by Mayhew [83]. Assessment of in 2 × 2 contingency table was performed separately for all different cell structures including plasmodesmata and tubular structures, cytoplasm and vacuole with the GraphPad Software [84], using the Fisher’s exact test. For a more extensive analysis, we also performed an odds ratio (OR) calculation as it was described by Mayhew and Lucocq [85].

## 5. Conclusions

Studies on pathogenic changes during viral plant-pathogen interactions are being conducted at different levels, from the whole-organ morphology to the tissues, cells and subcellular organelles, and the analyzes of cell structures have become increasingly common. These investigations show a range and complexity of pathogenesis during viral infection. PDV is an interesting and extremely damaging viral pathogen, distributed worldwide. Our multifaceted study combines the computer-assisted methods with the ultrastructural observations and the immunolocalization of CP and MP PDV proteins in order to better understand the pathogenesis and cell-to-cell transport of this virus. TEM ultrastructural analyses show the involvement of specific organelles including vacuole, ER, chloroplasts, and mitochondria in the PDV-infected cucumber tissue. PDV infection leads to sequential alterations in the infected cells. All results indicate that intercellular transport of PDV is similar to related *Bromoviridae* BMV and AMV.

## Figures and Tables

**Table 1:** Quantification of preferential double-immunogold localization of CP and MP in 2 × 2 contingency table from GraphPad Software. (A) Quantification of double-immunolocalization parameters in PDV-infected cucumber leaves. Two-tailed *p* value (*p*) for cell segments analyses was less than 0.0001. (B) Quantification of double-immunolocalization parameters in mock-inoculated cucumber leaves. No localization was observed. OR—odds ratio, CP_g20_+—presence of 20 nm gold particles associated with presence of CP epitope, CP_g20_−—absence of 20 nm gold particles associated with presence of CP epitope, MP_g10_+—presence of 10 nm gold particles associated with presence of MP epitope, MP_g10_−—absence of 10 nm gold particles associated with presence of MP epitope. In table bold and red color value is result of statistical analyses of Quantification of preferential double-immunogold localization.

Double-Immunolocalization Parameters				
**(A) PDV-infected mesophyll cells:**				
**-plasmodesmata and tubular structures**				
Protein	MP_g10_+	MP_g10_−	Row totals	Ratio MP_g10+_/MP_g10_−
CP_g20_+	47	6	53	7.83
CP_g20_−	13	20	14	0.65
Column totals	60	27	87	**OR = 12.04**
**-cytoplasm**				
Protein	MP_g10_+	MP_g10_−	Row totals	Ratio MP_g10+_/MP_g10_−
CP_g20_+	41	24	65	1.7
CP_g20_−	9	30	39	0.3
Column totals	50	54	104	**OR = 5.66**
**-vacuole**				
Protein	MP_g10_+	MP_g10_−	Row totals	Ratio MP_g10+_/MP_g10_−
CP_g20_+	20	16	36	1.25
CP_g20_−	12	23	34	0.52
Column totals	32	38	70	**OR = 2.40**
**(B) Mock inoculated mesophyll cells:**				
**-Plasmodesmata, tubular structures, cytoplasm and vacuole**				
Protein	MP_g10_+	MP_g10_−	Row totals	Ratio MP_g10_+/MP_g10_−
CP_g20_+	0	0	0	0
CP_g20_−	0	0	0	0
Column totals	0	0	0	0
